# Improved Diagnostic Multimodal Biomarkers for Alzheimer's Disease and Mild Cognitive Impairment

**DOI:** 10.1155/2015/961314

**Published:** 2015-05-28

**Authors:** Antonio Martínez-Torteya, Víctor Treviño, José G. Tamez-Peña

**Affiliations:** ^1^Cátedra de Bioinformática, Tecnológico de Monterrey, 64849 Monterrey, NL, Mexico; ^2^Departamento de Investigación e Innovación, Escuela de Medicina, Tecnológico de Monterrey, 64710 Monterrey, NL, Mexico

## Abstract

The early diagnosis of Alzheimer's disease (AD) and mild cognitive impairment (MCI) is very important for treatment research and patient care purposes. Few biomarkers are currently considered in clinical settings, and their use is still optional. The objective of this work was to determine whether multimodal and nonpreviously AD associated features could improve the classification accuracy between AD, MCI, and healthy controls, which may impact future AD biomarkers. For this, Alzheimer's Disease Neuroimaging Initiative database was mined for case-control candidates. At least 652 baseline features extracted from MRI and PET analyses, biological samples, and clinical data up to February 2014 were used. A feature selection methodology that includes a genetic algorithm search coupled to a logistic regression classifier and forward and backward selection strategies was used to explore combinations of features. This generated diagnostic models with sizes ranging from 3 to 8, including well documented AD biomarkers, as well as unexplored image, biochemical, and clinical features. Accuracies of 0.85, 0.79, and 0.80 were achieved for HC-AD, HC-MCI, and MCI-AD classifications, respectively, when evaluated using a blind test set. In conclusion, a set of features provided additional and independent information to well-established AD biomarkers, aiding in the classification of MCI and AD.

## 1. Introduction

Alzheimer's disease (AD) is the most common form of dementia, affecting more than five million people in the United States [1] and accounting for between 60% and 80% of the 44.35 million estimated worldwide dementia cases [2]. Its hallmark pathological lesions are abnormal brain deposits of *β*-amyloid (A*β*) plaques and neurofibrillary tangles formed by the hyperphosphorylated protein tau [3]. An early detection of AD allows prompt evaluation and treatment of reversible or treatable causes, management of symptoms with medication, inclusion in clinical trials, physicians, and caregivers to be aware of patients who may soon have difficulties and permits the affected person to plan ahead while they still have the capacity to make important decisions about their future care [1]. An established risk factor for AD is mild cognitive impairment (MCI), a condition in which subjects show problems with language, memory, or another cognitive ability [4]. Although the underlying cause of some MCI cases might not be AD [5], the progression from MCI to AD happens at a higher rate than that from an unaltered cognitive status [6], making MCI a primary endpoint in several randomized controlled trials [7–9] and MCI to AD progression the outcome of several studies predicting future cognitive decline [10–12].

The most used criteria for the clinical diagnosis of AD were established almost 30 years ago by the National Institute of Neurological, Communicative Disorders and Stroke and Alzheimer's Disease and Related Disorders Association (NINCDS-ADRDA) workgroup [13]. However, it has been reported to be inaccurate in up to 20% of cases, when performed in specialized research academic centers on patients in later stages studied over several years [14] and to have sensitivity and specificity ranging from 70.9 to 87.3% and from 44.3% to 70.8%, respectively [15]. Consequently, the criteria may lead to even more incorrect diagnoses in patients at earlier stages of the disease, particularly for those with MCI. Because of this, there has been a pressing need to improve the accuracy of diagnosis. It was expected that imaging and biological biomarkers could provide this improved accuracy [16], which resulted in two recent revisions of the NINCDS-ADRDA criteria, one by the National Institute of Aging (NIA) and Alzheimer's Association [17–19] and the other by Dubois et al. [20]. Both revised criteria now recommend the use of biomarkers to support AD and MCI due to AD diagnoses. However, only the five most widely studied biomarkers of AD were incorporated into the diagnostic criteria. The former revision indicates that biomarkers are meant to be used as complimentary to the paramount clinical diagnosis (i.e., not strictly needed to perform a clinical diagnosis of MCI and AD), and the latter strongly recommend their introduction to improve AD diagnosis, despite being only at research settings. Biomarkers being taken into account are low levels of the 42-amino-acid variant of A*β* (A*β*42) in cerebrospinal fluid (CSF), elevated CSF t-tau or p-tau (total or phosphorylated, resp.), abnormal tracer retention on amyloid positron emission tomography (PET) imaging, decreased fluorodeoxyglucose (FDG) uptake on PET, and atrophy on structural magnetic resonance imaging (MRI), the last three measured in a specific topographic pattern.

Multimodal biomarkers have shown to improve the accuracy of AD and MCI diagnosis and might also serve as indirect measures of disease severity [21–23]. However, the features being used to construct such biomarkers have been limited to include mainly those mentioned above. The use of these features comes from biomarker discovery studies involving univariate analyses guided by biological hypotheses. Nevertheless, studies have shown that multivariate biomarkers benefit from features not previously associated with AD on their own [24–27].

In this paper, we explore additional information from imaging sources (e.g., cortical thickness and hypometabolic convergence index), biological tests (e.g., complement component 3 concentrations and TOMM40 poly-T variable length), and clinical records (e.g., blood pressure, drug sensitivities, and presence of a depressed mood). Our objective was to determine whether multimodal and nonpreviously AD associated features could improve the classification accuracy between subjects with AD, MCI, and healthy controls (HC), building on preliminary versions [28].

## 2. Methods

### 2.1. Data

Data used in the preparation of this paper were obtained from Alzheimer's Disease Neuroimaging Initiative (ADNI) database (http://adni.loni.usc.edu/). The ADNI was launched in 2003 by the National Institute on Aging, the National Institute of Biomedical Imaging and Bioengineering, the Food and Drug Administration, private pharmaceutical companies, and nonprofit organizations, as a $60 million, 5-year public-private partnership. The primary goal of ADNI has been to test whether serial MRI, positron emission tomography (PET), other biological markers, and clinical and neuropsychological assessment can be combined to measure the progression of MCI and early AD. Determination of sensitive and specific markers of very early AD progression is intended to aid researchers and clinicians in developing new treatments and monitoring their effectiveness, as well as lessening the time and cost of clinical trials. The Principal Investigator of this initiative is Michael W. Weiner, M.D., VA Medical Center and University of California, San Francisco. ADNI is the result of efforts of many coinvestigators from a broad range of academic institutions and private corporations, and subjects have been recruited from over 50 sites across USA and Canada. The initial goal of ADNI was to recruit 800 subjects but ADNI has been followed by ADNI-GO and ADNI-2. To date, these three protocols have recruited over 1500 adults, ages 55 to 90, to participate in the research, consisting of cognitively normal older individuals, people with early or late MCI, and people with early AD. The follow-up duration of each group is specified in the protocols for ADNI-1, ADNI-2, and ADNI-GO. Subjects originally recruited for ADNI-1 and ADNI-GO had the option to be followed in ADNI-2. For up-to-date information, see http://www.adni-info.org/.

**Figure 1:**
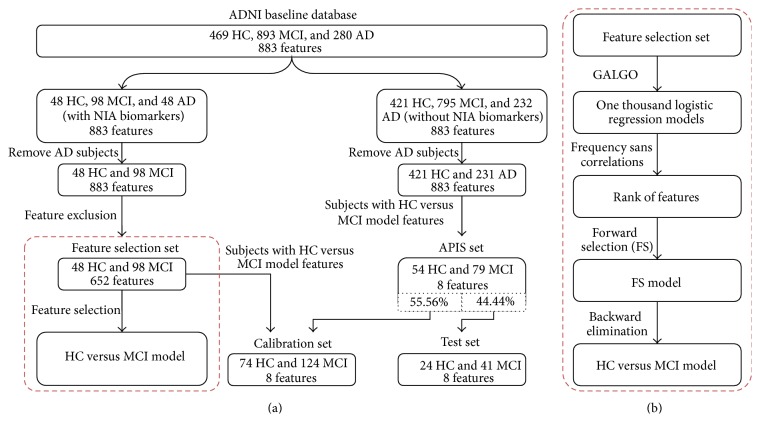
Workflow of the methodology for the Hc versus MCI analysis. (a) Summarized workflow for the HC-MCI analysis. The HC-AD and MCI-AD analyses follow the same workflow, removing the pertinent class of subjects. The red dashed rectangle shows the summarized feature selection methodology. (b) Extended feature selection methodology.

An overview of the overall methodology is shown in Figure 1. Available ADNI clinical and biological information up to February 2014 and features from MRI and PET analyses were analyzed. Information from neuropsychological questionnaires was not included in this study because diagnoses were partially based on some of them. The information obtained from biological samples included apolipoprotein E (APOE) genotyping, homocysteine and isoprostanes concentrations, urine and blood laboratory data (e.g., urine nitrite, monocytes, vitamin B12, and platelets), CSF laboratory data (i.e., red and white blood cell count, and glucose and protein results), rules-based medicine plasma data (e.g., interleukins, insulin, myoglobin, and thrombopoietin plasma concentrations), University of Pennsylvania (UPENN) CSF biomarker data (i.e., CSF concentrations of A*β*42, p-tau, and t-tau and the ratios of p-tau and t-tau to A*β*), UPENN plasma biomarker data (e.g., plasma concentration of A*β*42), and TOMM40 poly-T variable length data (i.e., length of each allele and mean, maximum, and minimum lengths) [30].

The MRI analyses from which information was acquired were the stroke summary analysis, reporting the number and location of strokes, and the white matter hyperintensity volume of the whole brain; the University of Arizona Gene Alexander Laboratory statistic parametric mapping voxel based morphometry analysis, reporting the mean gray matter value from 90 regions of interest (ROI); the University of California at San Diego Anders Dale Laboratory derived volumes analysis, reporting the volumes of 15 ROI; and the University of California at San Francisco FreeSurfer analysis, reporting the volume, surface area, and cortical thickness of 139 ROI [29].

The PET analyses from which information was obtained were the Banner Alzheimer's Institute analysis, reporting the globally normalized cerebral metabolic rate for glucose (CMRgl) in 70 ROI; the University of California at Berkeley Jagust Laboratory PET ROI analysis of glucose metabolism normalized to the pons, reporting the mean, median, mode, minimum, maximum, and standard deviation of FDG-PET from 5 different ROI; the University of Utah PET analysis, reporting the average CMRgl normalized to the pons in 3 ROI, and the number of pixels with hypometabolic activity that are two and three standard deviations below normal mean; and the New York University FDG-PET hippocampus analysis, reporting the mean FDG-PET of each hippocampus, normalized to the pons [31].

The information obtained regarding clinical data includes a symptoms checklist (e.g., insomnia, nausea, and depressed mood), family dementia history, own medical history (e.g., alcohol abuse, drug abuse, and allergies or drug sensitivities), a neurological exam (e.g., level of consciousness, motor strength, and presence of tremors), a physical exam (e.g., general appearance, presence of edemas and back pain), demography (e.g., age, gender, and ethnicity), and vital signs (e.g., temperature, pulse rate, and arterial pressure).

To guarantee that this study analyzed at least the biomarkers recommended by the revised criteria, subjects without biomarkers of A*β* accumulation and neuronal degeneration were excluded. The former was measured through CSF A*β*42 levels, and the latter by CSF t-tau and p-tau, hippocampal volume (obtained via MRI), and CMRgl in the posterior cingulate cortex (obtained via FDG-PET). Subjects without information from other sources previously found to be linked to AD in literature (i.e., maternal dementia history [32], white matter hyperintensity [33, 34], voxel based morphometry [35], and APOE genotyping [36, 37]), but not included by the revised criteria, were also excluded. All variables were *z*-standardized as defined by *z*_*ij*_ = (*x*_*ij*_ − *μ*_*j*_)/*σ*_*j*_, where *z*_*ij*_ and *x*_*ij*_ are the *z*-score and the raw measurement of the *i*th subject for the *j*th variable and *μ*_*j*_ and *σ*_*j*_ are the mean and the standard deviation of the entire ADNI population for the *j*th variable.

### 2.2. Feature Selection

Three binary classification analyses were performed to compare the three classes of subjects (HC-AD, HC-MCI, and MCI-AD). For each analysis, variables with missing data for more than 20% of the subjects of either class were excluded. The three analyses were executed similarly. First, an explorative search was performed using GALGO [38]. Briefly, it employs a multivariate feature selection strategy based on genetic algorithms that imitate natural selection during biological evolution. One thousand five-feature logistic regression models were obtained. Models evolved from an initial set of random models throughout 300 generations. In each generation, the fittest models reproduced, recombined, and mutated. The fitness was defined as the accuracy using a 3-fold cross-validation for the HC-MCI and MCI-AD analyses and 4-fold for the HC-AD, as suggested by GALGO's *k* optimization equation. At each cycle, subjects who did not have information on all features of the model being evaluated were not taken into account.

Features were then ranked according to their frequencies in the 1,000 regression models avoiding correlated features. For every pair of correlated features (Pearson *ρ* correlation coefficient larger than 0.8 at a *p* value smaller than 0.05), the least frequent was discarded, and its frequency was added to the most frequent feature. The ranked features were then used to generate a representative model with a customized forward selection (FS) strategy. The classical FS generates nested models, adding the next best ranked feature, one at a time, and selects the model that resulted in the maximum fitness. To avoid the inclusion of futile features, only those whose addition to its parent model resulted in a positive integrated discrimination improvement (IDI) [39] at a *p* value lower than 0.05, measured using the same *k*-fold sets as with GALGO, were included in the model. An example of this process is shown in Supplementary Figure 1 in the Supplementary Material available online at http://dx.doi.org/10.1155/2015/961314.

The final model was obtained after reducing the FS model with a backward elimination methodology. As shown in Supplementary Figure 2, during each cycle of this process, the IDI for the parent model and the same model after removing its terms, one at a time, was evaluated. The feature whose addition to its parent model resulted in the smallest IDI-related *z*-score was removed, provided that such a score was not significant (*p* value higher than 0.05). This process was carried on until no features could be removed using these criteria.

### 2.3. Validation Set

To validate the final model and to increase the population size, its features were used as a new filter. Subjects previously excluded from the study due to lack of data were examined, and those with information on the features of the final model were included in the validation study. For example, subjects without APOE4 data were originally removed from this study but were APOE4 not to be included in the final model; this subset was to be reconsidered for inclusion in the validation set. These subjects generated the* a posteriori* included subjects (APIS) set. The model was then calibrated using the population from the feature selection methodology and a random sample from the APIS set. Then, this calibrated model was tested in the remaining APIS population, the test set. The size of the sample from the APIS set included in the calibration set was defined so that a four to one proportion remained between such a set and the test set.

### 2.4. Statistical Analysis

The test set was used to evaluate the model for its sensitivity, specificity, accuracy, and area under the Receiver Operating Characteristic (ROC) curve (AUC). Sensitivity for the HC-AD and the MCI-AD subsets refers to the ratio of accurately predicted AD subjects to the total diagnosed AD subjects, and similarly for the HC-MCI subset, substituting AD with MCI. Additionally, the odds ratio of the magnitude of the regression coefficient at two standard deviations from the mean of the ADNI population was used to estimate the impact each feature had within the model. The calibration set was also used to evaluate the performance of the model, measuring its sensitivity, specificity, accuracy, and AUC using one thousand randomly generated bootstrap samples.

Lastly, to find out the probability of finding by chance a model with a similar performance, an additional experiment was performed. One thousand random models of the same size as the proposed model were generated from the feature selection set, and each one was evaluated using 1,000 bootstrap samples. The probability was estimated as the proportion of random models outperforming the proposed model to the total number of random models. The statistical analysis and all data handling were performed on R [40]; AUC values were obtained using the ROCR package [41].

## 3. Results

### 3.1. Data

The feature selection set resulted in a total of 48 HC, 98 MCI, and 48 AD subjects, and the calibration and test sets varied in size depending on the features from each model, since they were used to filter subjects. The demographic information of the three sets of subjects that were used in the methodology, per analysis, is shown in Table 1. It is important to notice that the demographic information is not based on all the subjects considered for each set. Instead, data from only those subjects who had information on all features of the final models were taken into account. Additionally, the HC-AD, HC-MCI, and MCI-AD datasets yielded 655, 652, and 799 features, respectively, after excluding those with a high missing data proportion.

### 3.2. Feature Selection

The most frequent features from the 1,000 genetic algorithm models in each analysis can be found in Supplementary Table 1. The features and corresponding coefficients that were included in the three resulting logistic regression models are shown in Table 2. Additional details on these features are included in Supplementary Table 2.

The HC-AD model contains three imaging features, two from MRI and one from PET analyses. The model included two well-established AD features, but also a novel feature, the surface area of the left superior frontal gyrus, having a significant coefficient and an odds ratio of 7.64. The model generated for the HC-MCI analysis had eight features, including biological, MRI, and medical history information. It included two well-known AD features, the ratio of CSF t-tau to A*β*42 and the volume of the left hippocampus. Interestingly, the relation of other 6 features with AD has not been widely investigated. These include the average cortical thickness of the right medial orbitofrontal cortex, with a 7.80 odds ratio, almost as large as the 8.58 odds ratio of the volume of left hippocampus. The MCI-AD model was built with seven biological and MRI features, none of which were NIA recommended biomarkers. In addition, we noted that no biological variables were needed to distinguish between HC and AD subjects, whereas in the transition from HC to MCI, the t-tau and red blood count played an important role. Similarly, three plasma proteins were important in the MCI to AD transition, having, in this model, the largest odds ratios (10.49 for the complement component 3, 7.01 for the monocyte chemotactic protein 4, and 4.30 for the apolipoprotein D).

Overall, all models included at least one feature with an odds ratio of ten or higher, but there were also four features that did not reach an odds ratio of two. As expected, most of the variables showed prominent differences between means, proportionally to their odds ratios.

### 3.3. Performance

**Figure 2:**
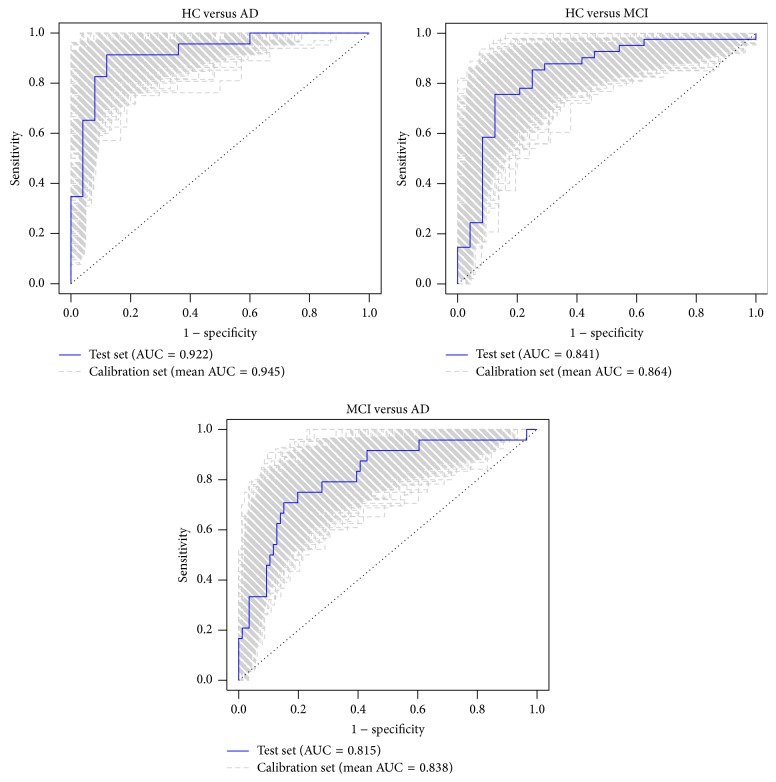
ROC curves for the calibration and test sets.

The accuracies, sensitivities, specificities, and AUC values of the three models are shown in Table 3. Although a decrease in the performance of the test sets with respect to their calibration sets was observed, accuracies and AUC values in the test sets lay within the 95% confidence interval of the their counterpart values in the calibration sets, as clearly shown in Table 3 and in the ROC curves shown in Figure 2.

**Figure 3:**
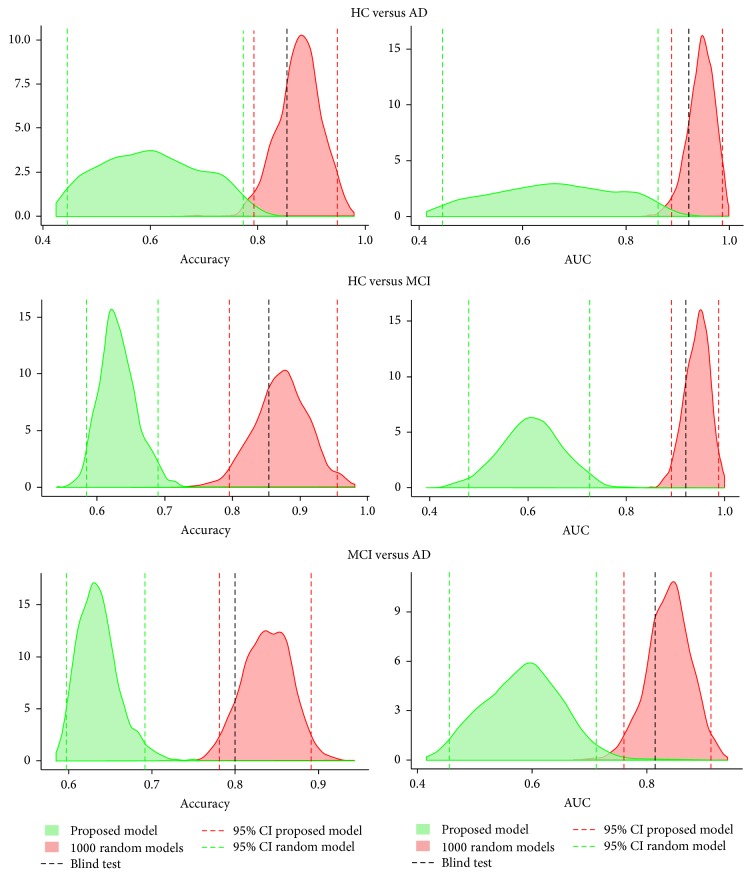
Accuracy and AUC of the proposed and random models. Red density distributions show the accuracy and AUC from the 1,000 bootstrap samples in the calibration set. Green density distributions show the accuracy and AUC from the 1,000 random models, each one evaluated using also 1,000 bootstrap samples in the calibration set. The black dashed line represents the accuracy and AUC evaluated in the test set.

Lastly, we tested whether the performance obtained was random by comparing it with the performance of 1,000 random models of the same length.  Figure 3 displays the density distribution of the accuracy and AUC achieved by the models in the calibration sets compared with the random models. It also shows the accuracy and AUC of the proposed models evaluated in the test set. The performance of the proposed models, evaluated in the calibration and test sets, was out of the 95% confidence interval of the performance of the random models. Consistently, the results from the test set lay within the confidence interval of the calibration set results.

## 4. Discussion

The results from this work evidence that HC, MCI, and AD subjects could be accurately classified using models generated through a feature selection methodology that explored a large multimodal database. More interestingly, they also demonstrate that some features currently not regarded as paramount for the diagnosis of AD and/or MCI due to AD may be relevant for such a task.

Given that this work was motivated to determine whether features not previously associated with AD might be important in the diagnosis of the disease, the use of as many features as possible was strived for. Because of this, the size of the database was very limited in size, since only a small amount of subjects had information on all the features being analyzed. The classification problem was binarized for the sake of detecting features that have a subtle link with cognitive decline at different stages of the disease, also avoiding the need to further reduce the size of the dataset. The main advantage of this stratification is that each model highlights specific features that may be obscured by the heterogeneity of the entire population. Therefore, three different models were designed to classify a specific set of classes, HC-AD, HC-MCI, and MCI-AD.

The performance of the models in the feature selection set evaluated with one thousand randomly generated bootstrap sample subsets seemed to show that models were not random and that interesting novel features might be useful. When the linear regression coefficients were tuned to this set of subjects and the model was evaluated in the whole APIS set, 3 out of 12 performance metrics (the accuracy and AUC of the HC-MCI model and the AUC of the MCI-AD model) were below the 95% confidence interval of the bootstrap results (Supplementary Table 3). These were thought to be the result of an overfitting effect, mainly due to the small size of the feature selection set, not being able to account for the variation found in the APIS set. This was prominently observed in the MCI-AD analysis, where the model was trained using the 132 subjects (89 controls) from the feature selection set, while the APIS set had 306 subjects (154 controls), as seen in Supplementary Table 4. So, clearly, there was an undersampling effect in the feature selection step.

In an attempt to soften this effect, a calibration set with all the subjects from the feature selection set and some randomly selected subjects from the APIS set was created. By doing so, the number of subjects used to calibrate the model was augmented, though at the cost of reducing the size of the set used to test the model. Tuning the coefficients using the calibration set resulted in a penalization thereof, compared to the coefficients obtained when calibrating using only the feature selection set. All coefficients were reduced in magnitude, even having the coefficient of one feature from the HC-MCI model practically reduced to zero. However, none of the coefficients had a change in sign, meaning that the effect of the feature detected by the feature selection algorithm in the reduced set was conserved.

Additionally, the results obtained with the test set not only lay between the 95% confidence interval of the bootstrap results, as shown in Figure 3, but also were better than the ones obtained when using the whole APIS set. This indicated that, by augmenting the size of the set used to calibrate the model, the undersampling effect was reduced without dismissing the selection of features done on a subset. In Figure 3, it can also be seen that the accuracy and AUC obtained by proposed combinations of features were unlikely due by chance. As expected, the HC-AD model resulted in the best performance since the HC and the AD populations have the most cognitively dissimilar subjects. The results from the other two models were also promising, both achieving an AUC higher than 0.8.

Regarding the biomarkers of A*β* accumulation and neuronal degeneration included in the revised criteria, they were all present in the models, except for the abnormal tracer retention on amyloid PET imaging, which was not included in this study due to lack of sufficient data. Furthermore, whenever these biomarkers were present in a model, they had the highest odds ratios, meaning that they were the most relevant risk factors for either MCI or AD. This result provides additional support that our methodology is able to find relevant features. The volume of the left hippocampus, which was highly correlated to its right hemisphere counterpart (Pearson *ρ* = 0.88 for HC-AD and Pearson *ρ* = 0.86 for HC-MCI), stood out by aiding in distinguishing HC from both MCI (odds ratio = 8.58) and AD subjects (odds ratio = 273.11), reinforcing the idea that hippocampal volume is a very important risk factor for AD. Decreased FDG uptake on PET, measured via the globally normalized CMRgl from the left angular gyrus, was useful in discriminating between HC and AD subjects (odds ratio = 56.59), meaning that the HC-AD model had information of both A*β* accumulation and neuronal degeneration. And finally, t-tau and A*β*42 aided in the differentiation of HC and MCI subjects through their ratio (odds ratio = 25.97). This result is consistent with the AD pathological cascade, which indicated that CSF tau concentrations are already abnormal in MCI (due to AD) subjects and that A*β* accumulation starts happening even before any signs of cognitive decay appear [3].

Even though some of these biomarkers were highly ranked, none were present in the MCI-AD model, suggesting that they do not provide additional information once the other features found are included in the model. This is particularly relevant, considering that biomarkers of neuronal degeneration should show an important difference between MCI and AD subjects according to the aforementioned pathological cascade. In this model, the feature with the highest odds ratio was the plasma concentration levels of complement component 3, a protein whose activation products are localized with A*β* deposits in the brain of AD subjects and which is thought to play a crucial role as mediator between the A*β* deposits and the inflammatory response leading to neurotoxicity [42]. This protein has already been linked to MCI and AD [43, 44]. The plasma concentration level of apolipoprotein D was also found in this model, a feature whose increased levels have also been linked to AD [45]. Interestingly, to our knowledge, no association has been made between AD and the plasma concentration levels of monocyte chemotactic protein 4, which has an odds ratio of 7.01 in the model. This suggests that the role of such feature in the pathological cascade of AD, if any, should be further investigated. The TOMM40 poly-T variable length was initially included in the model but its coefficient was reduced to zero once the subset of the APIS set was included in the calibration.

On the other hand, novel features were also included in models alongside recommended biomarkers. The surface area of the left superior frontal gyrus, a key component of the neural network of working memory [46], was found in the HC-AD model. This feature was highly correlated to the surface area of the left hemisphere (Pearson *ρ* = 0.84) and of the right hemisphere (Pearson *ρ* = 0.81), which may lead to thinking that the biological effect being measured by this feature could be happening in the whole brain, but in a more intense way in this particular brain region. This feature is particularly interesting because, in this model, it is found alongside the CMRgl of the left angular gyrus and the volume of the hippocampus, biomarkers of A*β* accumulation, and neuronal degeneration, respectively. Thus, this novel feature is providing independent information to the one provided by the NIA recommended biomarkers. It highlights that there is information currently not being taken into account that could be used to improve the accuracy of diagnosis.

A similar conclusion could be reached from the HC-MCI model, which also includes biomarkers of A*β* accumulation and neuronal degeneration, and six additional novel features. However, in this case, A*β* accumulation is not being measured through one of the NIA recommended biomarkers, but through the ratio of t-tau and A*β*42. From these six novel features, the two with the largest odds ratio are measurements of cortical thickness, one from the right medial orbitofrontal cortex and the other from the right temporal lobe. The potential of cortical thickness for the classification of AD has already been proposed [47–49], and thus it is of no surprise that such features could aid in the classification of HC and MCI subjects. However, the fact that the information provided by these features adds to the information provided by the NIA recommended biomarkers enhances the putative importance of studying these kinds of measurements.

Another advantage of this methodology resides in having models with small numbers of features, which are not necessarily statistically significant on their own. After a Bonferroni correction to the *p* value of the Wilcoxon rank-sum test, performed in every feature with a frequency different than zero, 52 features were statistically significant in discriminating between HC and AD subjects, the most significant feature being the ratio of CSF t-tau to A*β*42. Were only significant features to be considered, the novel feature from the HC-AD model would not be taken into account. Using a univariate logistic regression model with the most significant feature, trained in the calibration set and evaluated in the test set, an accuracy of 0.800 and an AUC of 0.857 were achieved, in comparison with the 0.854 and 0.922 values obtained using the proposed model.

The main limitation of this study was the lack of amyloid PET imaging information, one of the NIA recommended biomarkers. An analysis of the correlation between the novel features found in the proposed model and such information should be performed to verify that these novel features are indeed independent of A*β* accumulation and provide additional information. Importantly, nearly perfect concordance is present between abnormally low CSF A*β*42 and positive amyloid PET imaging in subjects who have undergone both tests [50, 51], which led us to believe that the results from such an experiment would not change the conclusions reached in this study. This study was also limited by differences in the provided data.

## 5. Conclusion

HC, MCI, and AD subjects were accurately classified through models generated via a feature selection methodology that searched a large multimodal database. The models included some NIA recommended biomarkers of A*β* accumulation and neuronal degeneration and novel features that provided independent information. Consequently, the current diagnostic criteria for AD and MCI due to AD could be enhanced by adding information from other sources. Features not previously related to AD should keep being investigated.

## Supplementary Material

The Supplementary Material includes tables and figures that allow for a better understanding of the methodology and the obtained results. Supplementary Table I describes the top ranked features for each experiment, highlighting those who were included in the final models; Supplementary Table II details the studies from which the features found in the final model were obtained; Supplementary Table III shows the performance of the models when tuned using the feature selection set, and evaluated in the APIS set; and Supplementary Table IV characterizes these populations. Supplementary Figure 1 and Supplementary Figure 2 exemplify the forward selection and backward elimination methodologies, respectively.

## Figures and Tables

**Table 1:** Demographics of the study group.

Analysis	Cohort	Class	Subjects (female proportion)	Mean age (SD)	Mean years of schooling (SD)
HC-AD	Feature selection set	HC	48 (37.5%)	74.2 (5.2)	15.6 (3.2)
		AD	48 (37.5%)	74.6 (7.6)	14.6 (3.7)
	Calibration set	HC	75 (34.7%)	74.4 (4.9)	16 (3.1)
		AD	70 (38.6%)	74.9 (7.1)	14.5 (3.4)
	Test set	HC	25 (48%)	75.8 (4.1)	15.5 (3.3)
		AD	23 (47.8%)	74.5 (8)	15.1 (2.9)

HC-MCI	Feature selection set	HC	44 (40.9%)	73.9 (5.2)	15.6 (3.2)
		MCI	86 (31.4%)	74.5 (7)	15.8 (3)
	Calibration set	HC	74 (50%)	74.6 (5.3)	15.5 (3)
		MCI	124 (32.3%)	74.3 (6.9)	15.6 (3)
	Test set	HC	24 (62.5%)	73.8 (4)	16.1 (2.3)
		MCI	41 (39%)	72.9 (7.9)	15.9 (3.3)

MCI-AD	Feature selection set	MCI	89 (30.3%)	74.2 (7.2)	16 (3)
		AD	43 (37.2%)	74.5 (7.6)	14.6 (3.8)
	Calibration set	MCI	257 (33.5%)	74.1 (7.1)	15.7 (3.1)
		AD	71 (46.5%)	74.4 (7.1)	14.7 (3.4)
	Test set	MCI	86 (37.2%)	72.9 (7.4)	15.9 (3)
		AD	24 (37.5%)	72.8 (10)	16 (2.7)

SD stands for standard deviation.

**Table 2:** Resulting models and characteristics of each feature.

Analysis	Feature	Coefficient	OR	Mean (SD)	
				Controls	Cases
HC-AD	Volume of left hippocampus	−2.8^*∗∗∗*^	273.11	0.52 (0.72)	−0.66 (0.82)
	Globally normalized CMRgl from left angular gyrus	−2.02^*∗∗∗*^	56.59	0.5 (0.66)	−0.64 (1.15)
	Surface area of left superior frontal gyrus	1.02^*∗∗*^	7.64	−0.01 (0.82)	0.11 (0.99)

HC-MCI	Ratio of CSF t-tau to A*β*42	1.63^*∗∗∗*^	25.97	−0.54 (0.39)	0.16 (0.95)
	Volume of left hippocampus	−1.07^*∗∗∗*^	8.58	0.47 (0.59)	−0.17 (0.82)
	Standard deviation of the cortical thickness of the right temporal lobe	0.72^*∗∗*^	4.23	−0.49 (0.8)	0.11 (1.01)
	Red blood cell count	0.15	1.34	−0.05 (0.71)	0.16 (1.37)
	SPM VBM measure of the 4th and 5th vermal lobules	0.53^*∗*^	2.91	−0.03 (0.97)	0.26 (0.97)
	Average cortical thickness of right medial orbitofrontal cortex	−1.03^*∗∗∗*^	7.8	0.54 (0.75)	−0.13 (0.86)
	Surface area of left temporal pole	0.31	1.86	−0.36 (0.78)	−0.24 (0.89)
	Whether the subject has suffered from endocrine-metabolic diseases	0.36	2.07	−0.24 (0.93)	−0.12 (0.98)

MCI-AD	Plasma concentration levels of complement component 3	1.18^*∗∗∗*^	10.49	0.01 (0.97)	0.79 (1)
	SPM VBM measure of the right middle temporal gyrus	−0.61^*∗∗*^	3.41	0.06 (1.03)	−0.47 (0.95)
	Sum of both alleles TOMM40 poly-T variable length	0	1.01	0.04 (0.99)	0.04 (0.92)
	Standard deviation of the cortical thickness of “left unknown” region	−0.72^*∗∗∗*^	4.23	0.02 (0.88)	−0.56 (0.85)
	Plasma concentration levels of monocyte chemotactic protein 4	−0.97^*∗∗∗*^	7.01	0.16 (0.93)	−0.23 (0.83)
	Plasma concentration levels of apolipoprotein D	0.73^*∗∗∗*^	4.3	−0.15 (1.03)	0.33 (0.9)
	Surface area of right lateral orbitofrontal cortex	0.32	1.9	−0.11 (0.93)	−0.11 (0.97)

Coefficients, odds ratios (OR), and *p* values were obtained using the calibration set. The “left unknown” region was defined also by the University of California at San Francisco FreeSurfer analysis group [29]. Control refers to HC subjects for the HC-AD and HC-MCI analyses and to MCI for the MCI-AD analysis. *∗∗∗*, *∗∗*, and *∗* symbols indicate a probability lower than 0.001, 0.01, and 0.05, respectively, for the logistic regression coefficient being worth zero.

**Table 3:** Model performance.

Analysis	Cohort	Accuracy	Sensitivity	Specificity	AUC
HC-AD	Calibration set	0.877 (0.792–0.948)	0.849 (0.696–0.964)	0.905 (0.75–1)	0.945 (0.889–0.987)
	Test set	0.854	0.913	0.8	0.922

HC-MCI	Calibration set	0.802 (0.718–0.877)	0.862 (0.75–0.957)	0.704 (0.531–0.875)	0.864 (0.789–0.934)
	Test set	0.785	0.805	0.75	0.841

MCI-AD	Calibration set	0.838 (0.781–0.892)	0.476 (0.281–0.68)	0.941 (0.88–0.989)	0.838 (0.76–0.911)
	Test set	0.8	0.333	0.93	0.815

Calibration set results represent the mean of the 1,000 bootstrap samples and the values in parenthesis represent the 95% confidence intervals.
